# Soothing Properties of Glycerol in Cough Syrups for Acute Cough Due to Common Cold

**DOI:** 10.3390/pharmacy5010004

**Published:** 2017-01-20

**Authors:** Ronald Eccles, Pascal Mallefet

**Affiliations:** 1 Common Cold Centre, Cardiff School of Biosciences, Cardiff University, Cardiff CF10 3AX, UK; 2 Novartis Consumer Health SA, a GSK consumer Healthcare Company, 1260 Nyon, Switzerland; pascal.x.mallefet@gsk.com

**Keywords:** acute cough, cough syrups, glycerol

## Abstract

The treatment and management of acute cough due to common cold costs billions of dollars of healthcare expenditure and there is a growing opinion that a simple linctus containing glycerol with flavourings such as honey and lemon is a safe and effective treatment for acute cough in children and adults. Glycerol is a component of most cough syrups, and although it is often thought of only as a solvent or thickening agent in cough syrups, it may be a major component for the efficacy of cough syrups due to its special properties of lubrication, demulcency, sweetness, and acting as a humectant. The major benefit of cough syrups in soothing cough is likely due to the properties of the syrup rather than the active ingredients and this review discusses the special properties of glycerol in relation to the treatment of acute cough.

## 1. Introduction

Glycerol, referred to as glycerine or glycerin in the United States, is one of the most versatile and commonly used chemicals in the pharmaceutical, cosmetic and chemical fields and it is quoted as having over 1500 known end uses [1,2]. Glycerol is found naturally in all living cells in the form of triglycerides and it occurs naturally in wines, beers, breads and most products of fermentation [3]. Quoting C.E. Gentry, Procter & Gamble Co. (Cincinnati, OH, USA) glycerol product manager in 1984, “Glycerol may be the most versatile chemical known to man. It is used to make glue to stick things together, and in dynamite to blow things apart. It is used in cough suppressants and suppositories. Glycerol is used in hair sprays and house paint. It is an ingredient in expensive liqueurs and cheap pet foods” [4]. Glycerol is a component in most cough syrups. It is often thought of only as a solvent or thickening agent; however, it also contributes to the efficacy of the cough treatment due to its special properties of lubrication, demulcency, sweetness, and acting as a humectant.

This short review will focus on the role of glycerol as an important component for the efficacy of cough syrups.

## 2. Special Properties of Glycerol

**Figure 1:**
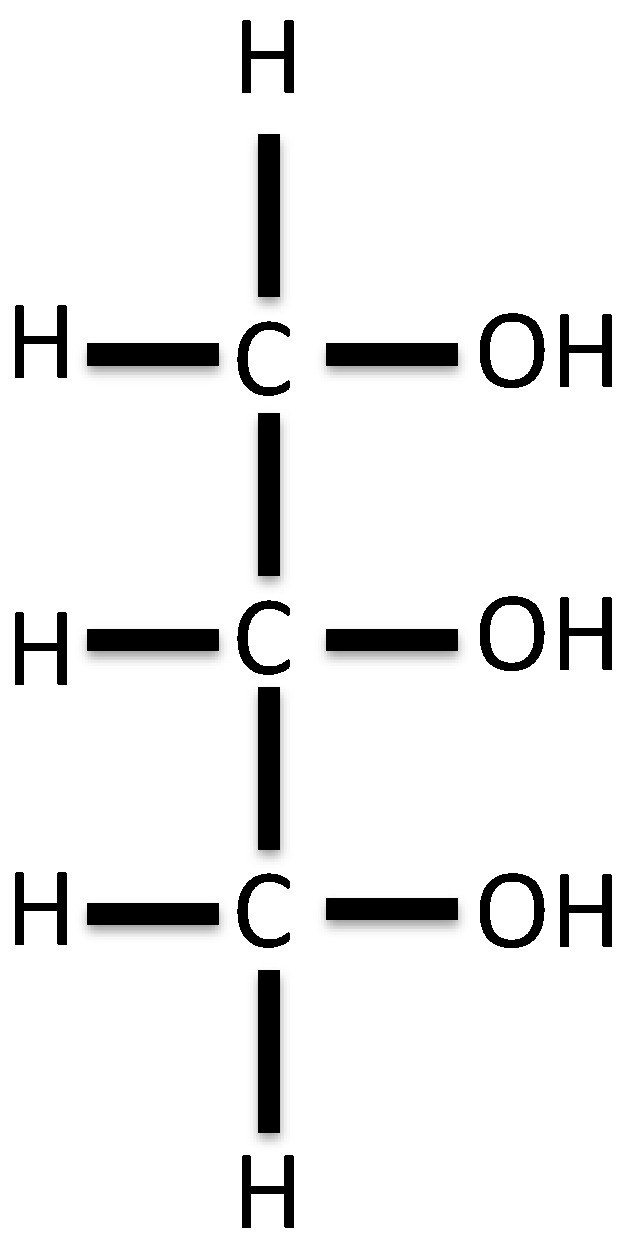
Chemical structure of glycerol.

Glycerol (1,2,3-propanetriol) as shown in Figure 1 is a colourless, odourless, viscous liquid with a warm sweet taste.

Glycerol is obtained from natural sources such as animal fats and also as a side product of the soap and petrochemical industries. In its pure anhydrous state, glycerol has a specific gravity of 1.261 g·mL^−1^, a melting point of 18.2 Centigrade, and a boiling point of 290 Centigrade [2,3]. Glycerol contains three hydrophilic alcoholic hydroxyl groups which make it very soluble in water and also account for its hygroscopic properties and behaviour as a humectant. Humectants are substances that retain moisture and in turn give softness, and this hygroscopic property of glycerol makes products containing glycerol, such as lotions and syrups, soft, flexible, and creamy [3]. Glycerol is added to many products because of its ability to attract and hold water and this is the part of its humectant property that is so useful in cosmetics, especially skin products, which are marketed for their moisturizing action [1]. The humectant property of glycerol may at first seem at odds with its moisturizing action, but it is the property of glycerol to hold water close to the skin that provides the moisturizing effect and not the withdrawal of water from skin tissue [1].

Glycerol contributes a plasticizing effect, as glycerol and water act together to promote softness and flexibility, and a hydrating effect is achieved as the glycerol solution prevents drying out. This plasticizing or humectant effect is beneficial for products such as cosmetics, creams, lotions, tooth pastes, candy, cough drops and cough syrups [3]. Because of its hydroxyl groups, glycerol has solubility characteristics similar to those of water and simple aliphatic alcohols and this makes it a very useful solvent for cough medicines. Glycerol is viscous and has a high viscosity and this makes it useful as a thickening or bodying agent which gives viscosity to cough syrups. At normal temperatures, glycerol is a stable viscous liquid right up to concentrations of 100% without any crystallization. Glycerol is also a lubricant as it decreases the friction between moving surfaces, and for this reason, glycerol is used as a lubricant in artificial tears and also as an industrial lubricant in air compressors [2].

Glycerol has demulcent properties with the term demulcent meaning soothing (derived from the Latin *demulcere,* “caress”). A demulcent is a substance that forms a soothing film over a mucous membrane to relieve minor pain and inflammation of the membrane [5]. The demulcent property of glycerol is particularly useful when it is a component of artificial tears [6] and it contributes to the efficacy of cough syrups [7,8].

Glycerol is virtually non-toxic in the digestive system and non-irritating to the skin and sensitive membranes, except in very high concentrations when a dehydrating effect is apparent [3].

## 3. Acute Cough

Acute cough is commonly caused by viral infections of the upper respiratory tract (URTI) [9]. Cough is normally a protective reflex to prevent the entry of food and fluid into the respiratory tract, but viral infections then cause inflammation of the upper airways and a sensation of irritation with an urge to cough which results in coughing that is a nuisance rather than a benefit to the subject [9,10]. The sensation of irritation that causes the urge to cough is due to a hypersensitivity of the sensory nerves in the upper airway found mainly in the larynx. Cough can also be initiated by pharyngeal stimulation [11] and this indicates that sensory nerves in the pharynx may also be involved in the generation of cough associated with URTI, as pharyngitis is a common symptom associated with URTI [12].

## 4. Mechanism of Action of Glycerol in Soothing Cough

The major effect of cough syrups in soothing cough is due to the properties of the syrup rather than the active ingredients such as dextromethorphan. Up to 85% of the benefit of cough syrups may be due to the physical and chemical effects of the syrup which contribute to its demulcent action [7].

Glycerol is a component of many cough syrups, especially in honey and lemon syrups where the concentration of glycerol is usually around 0.75 g per 5 mL and where glycerol may be declared as the sole active ingredient of the cough syrup.

The efficacy of glycerol must be related to its physical and chemical properties, as glycerol does not have any known pharmacological actions.

### 4.1. Demulcent

Glycerol is often referred to as having demulcent properties [8,13] and it is commonly used as an emollient in skin products to soften and soothe skin [3]. Glycerol may work as a demulcent in the pharynx by coating and lubricating the pharyngeal surface. The moisturizing properties of glycerol may also help to soothe inflamed mucosal surfaces in the pharynx.

### 4.2. Lubrication

Glycerol has lubricant properties [8] and is used as a lubricant in machinery such as air conditioners and compressors [2] and in lubricating cosmetic gels and creams [14]. The lubricant properties of glycerol are beneficial in lubricating the pharyngeal area, as the surfaces of the pharynx and tongue slide over each other during swallowing and speech and this mechanical stimulation will irritate sensory nerves that trigger cough. Glycerol will coat the pharynx and act as a lubricant and reduce the friction between moving surfaces of the pharynx and tongue and therefore sooth cough. Glycerol may also influence the structure of the protein surface of the pharynx by decreasing the volume of proteins, and this may contribute to smoothing the surface and a lubricant effect [15].

### 4.3. Sweetness

Glycerol has a sweet, warm taste and is about 0.6 to 0.8 times as sweet as sucrose [13,16]. The sweetness of cough syrups has been proposed to be a major factor in their efficacy [17]. The sweet taste in cough syrups has been proposed to modulate cough at the level of the nucleus tractus solitarius, possibly by influencing the production of endogenous opioids [17]. This mechanism for an antitussive action of the sweet taste has been supported experimentally by demonstrating that rinsing the mouth with a sweet-tasting solution inhibits capsaicin-induced cough, whereas rinsing with a bitter-tasting solution had no effect on the cough threshold [18,19].

## 5. Discussion

The treatment and management of cough costs billions of dollars of healthcare expenditure and a huge burden in terms of days lost from school and loss of work hours [20]. For example, in the UK, acute cough resulted in an estimated cost of £875 million due to loss of productivity and a cost of £104 million to the healthcare system along with the purchase of non-prescription medicines [21]. Estimating the global prevalence of cough is difficult considering its benign and self-limiting nature. An estimated 48 million cases of acute cough occur every year in the United Kingdom [21].

In general, over-the-counter (OTC) cough suppressants for acute cough show inconsistent, variable and conflicting results in terms of clinical improvement and have limited efficacy for symptomatic relief [22,23,24]. Various concerns about their safety include unintentional consumption, medication overdose and an additive effect due to the intake of cough and cold medications with the same active ingredients, for example two multi ingredient medicines which both contain paracetamol. Improper use of OTC cough and cold medication has also resulted in fatalities in children [25,26,27]. Considering these points, health authorities such as MHRA, Health Canada and other professional health bodies do not recommend their use in children below the age of six and the FDA does not recommend the use of these products in children below four years of age [22,25,26,27,28,29]. Instead, several health authorities such as MHRA promote the use of a simple cough syrup or linctus containing glycerol, lemon, and honey, which are soothing, safe and generally cheap [27].

There is, at present, no published research on the efficacy of glycerol as a cough treatment and there is a need for randomised placebo-controlled clinical trials to determine the contribution of this common ingredient in cough medicines to the overall benefit of cough medicines.

## 6. Conclusions

Acute cough is a common condition and there is a growing opinion that a simple linctus containing glycerol with flavourings such as honey and lemon is a safe and effective treatment for cough in children and adults.
